# Supplementary material for: The interactome of intact mitochondria by cross-linking mass spectrometry provides evidence for coexisting respiratory supercomplexes
Source: Mol Cell Proteomics. 2017 Dec 8;17(2):216–32. doi: 10.1074/mcp.RA117.000470 (PMC5795388; doi:10.1074/mcp.RA117.000470)
Supplement: Supplemental Data [file supp_RA117.000470_133922_0_supp_23978_fzffwf.zip › spectra_annotation/mito_DR_spectra_annotation/465-1-3-1-31-1.pdf]

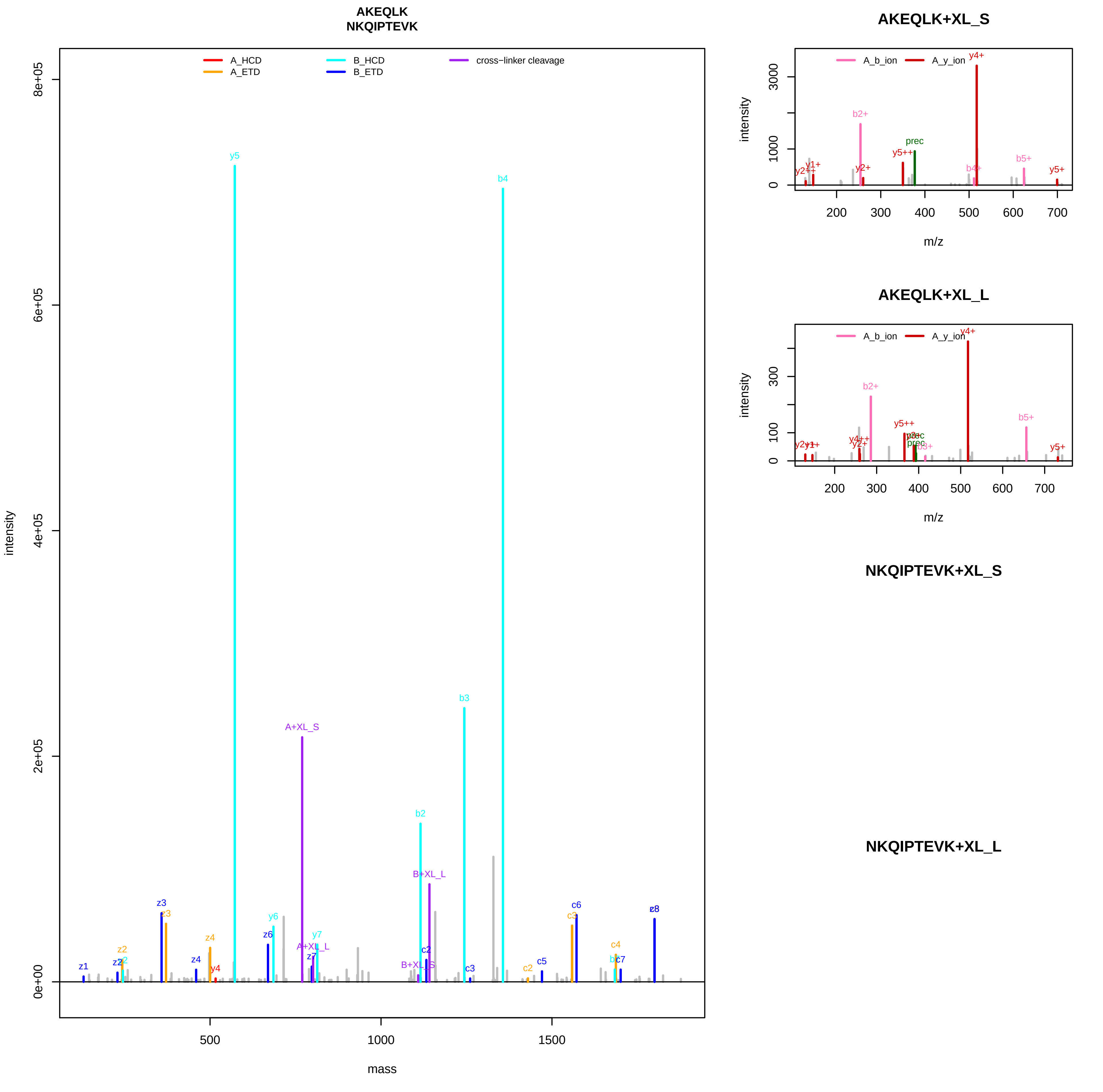

AKEQLK
NKQIPTEVK
A_HCD
A_ETD
B_HCD
B_ETD
cross−linker cleavage
intensity
mass
0e+00
2e+05
4e+05
6e+05
8e+05
500
1000
1500
y5
b4
b3
b2
A+XL_S
B+XL_L
A+XL_L
B+XL_S
y6
y7
z1
z2
z3
z4
z6
z7
y4
c2
c3
c4
c5
c6
c7
c8
AKEQLK+XL_S
A_b_ion
A_y_ion
b2+
b4+
b5+
y1+
y2+
y2++
y4+
y5+
y5++
prec
m/z
AKEQLK+XL_L
b3+
y4++
NKQIPTEVK+XL_S
NKQIPTEVK+XL_L
